# Simulation of resources use and abiotic stress management in various maize-based cropping systems

**DOI:** 10.3389/fpls.2025.1743567

**Published:** 2026-01-28

**Authors:** Khalid Hussain, Thomas Hilger, Erdoğan Eşref Hakki, Ayesha Ilyas, Sait Gezgin, Chalermchart Wongleecharoen

**Affiliations:** 1 Department of Soil Science and Plant Nutrition, Selcuk University, Konya, Türkiye; 2 Department of Agronomy, University of Agriculture, Faisalabad, Pakistan; 3 University of Hohenheim, Institute of Agricultural Sciences in the Tropics (Hans-Ruthenberg-Institute), Stuttgart, Germany; 4 Kasetsart University, Department of Soil Science, Bangkok, Thailand

**Keywords:** abiotic stress simulation, crop modeling, cropping systems, resource use efficiency, stable isotope discrimination, TDR profiling

## Abstract

Abiotic stress and low resource-use efficiency are among the main challenges in agricultural production systems. Stress management is key to sustainable production. It is still challenging to identify and manage prevailing stresses under field conditions due to limited knowledge of the mechanisms of multiple abiotic stressors in crops. Crop models are becoming popular in agriculture because of their diversified nature in identifying multiple abiotic stresses and resource-use management in the complex nature of agricultural production systems. This study combined field measurements and crop modeling to improve the understanding of below- and above-ground resources use (water, nutrients, and light) and their impact on crop productivity and stress management under various planting conditions. A two-year field trial was conducted in the Thai uplands, comparing six treatments: (T1) maize sole crop with tillage and fertilization; (T2) maize-chili intercropping with tillage and fertilization; (T3) same as T2 but with minimum tillage and *Canavalia ensiformis* relay cropping; (T4) same as T3 plus *Leucaena* hedgerows; (T5) same as T3 without fertilization; and (T6) same as T4 without fertilization. The Water Nutrient and Light Capture in Agroforestry Systems (WaNuLCAS) model was calibrated using data from T1, T4, and T6 and evaluated against independent observations from T2, T3, and T5. Row-wise aboveground biomass, grain nitrogen (N) and phosphorous (P) concentrations, δ¹³C values, soil volumetric water content, and root length density were measured over two growing seasons. Grain δ¹³C values were significantly less negative in rows near the hedge (−10.33‰) than in distant rows (−10.64‰). More negative grain δ¹³C values (−9.32‰, p ≤0.001) were observed in T6. Both field observations and model simulations showed reduced maize biomass and lower grain N and P concentrations in rows closest to the hedgerows, driven by root competition for nutrients. Soil moisture was consistently higher in intercropped systems, and hedgerow height control prevented shading, indicating no water or light limitations. From the results it is concluded that WaNuLCAS model accurately reproduced spatial biomass patterns (*EF = 0.95*, *RMSE = 0.98*, and *R2 = 0.96*) and correctly identified nitrogen and phosphorus stress in maize rows planted closely with leucaena hedgerows. Scenario simulations demonstrated that balanced increases in both N and P inputs most effectively alleviated nutrient competition and improved the long-term system productivity. This integrated field-model approach provides a robust framework for diagnosing resource competition and optimizing nutrient management in hedgerow-based agroforestry systems under upland conditions.

## 1 Introduction

Maize (*Zea mays* L.) is a major cash crop worldwide. The global maize market was valued at approximately 153 billion USD in 2025 and is expected to expand up to 215 billion USD by 2033, with an annual growth rate of approximately 2.4% (Vieira et al., 2025; Maize Market Report, 2025). In Thailand, the poultry sector alone consumes more than 8.2 million tons of maize, accounting for nearly 80% of the total national production, and approximately 70% of it is produced by smallholder farmers in the rainfed uplands of Thailand (Hayward, 2018; Pravalprukskul et al., 2023). The cultivation area of maize has increased and moved into marginal highland landscapes, many of which overlap with areas reserved for national forests or invade established forests (Hayward, 2018; Yap et al., 2017; Pravalprukskul et al., 2023). These upland agricultural systems are typically characterized by steep slopes, erosion-prone soils, and continuous cultivation of low-canopy crops, such as maize, which accelerate soil organic matter decline and expose soils to intensive surface erosion. In northern Thailand, nearly one-third of the total upland agricultural areas are now reported to suffer severe soil loss, illustrating the urgent need to adopt soil conservation practices (Pravalprukskul et al., 2023; Pakoksung, 2024).

Many soil conservation strategies, such as contour hedgerows, vegetative strips, residue retention, minimum tillage, and crop diversification, are being promoted to stabilize these degraded uplands, reduce soil loss, and improve long-term soil function (Talukdar et al., 2023). Among these soil conservation strategies, contour hedgerow intercropping can significantly reduce water runoff and soil erosion in sloping agricultural landscapes (Hussain et al., 2020; Yang et al., 2021). However, the introduction of hedgerows into annual crop systems may alter resource distribution. Competition for above- and belowground resources, particularly water and nutrients, may occur between trees and adjacent crops (Hussain et al., 2016; Montejo-Martínez et al., 2020; Zhao et al., 2025), whereas hedgerow shade may influence light capture depending on canopy height and management (Ladux et al., 2024; Raskin, 2025). In contrast, some contradictory studies have indicated that the hedgerows may enhance nutrient cycling, contribute substantial organic matter inputs, and improve soil structural stability, generating facilitative effects for the associated crops (Sanchez and McCollin, 2015; Montgomery et al., 2020; Worku and Mekoya, 2025). These contrasting interactions commonly result in spatial gradients of crop performance, with rows planted close to the hedgerows experiencing different resource environments than those located away from the hedges.

Currently, crop models are gaining popularity as modern tools can be helpful in understanding resource capture dynamics across diversified cropping conditions and can analyze such spatially differentiated interactions (Khongdee et al., 2022; Banerjee et al., 2024). Dynamic, process-based crop models can provide a useful framework for exploring how soil properties, management practices, and plant interactions influence system outcomes under varying environmental conditions (Wajid et al., 2021; Banerjee et al., 2024). The WaNuLCAS (Water, Nutrient, and Light Capture in Agroforestry Systems) model is specifically designed to analyze crop hedgerow interactions by representing root distribution and plant-available resource competition (Bhardwaj et al., 2024). Earlier applications of WaNuLCAS have successfully simulated nutrient redistribution, soil conservation effects, and crop performance under agroforestry and hedgerow-based systems (Hussain et al., 2016; Mahato and Kadaverugu, 2024), but field observations and validation were limited.

Our current study emphasizes the value of integrating field experimentation with process-based dynamic modeling to understand how abiotic stresses regulate crop performance and resource-use efficiency in diversified production systems. Such integrated approaches are particularly relevant where water, nutrient, and light dynamics vary sharply within production systems. In this study, we combined two consecutive year field observations with WaNuLCAS simulations to investigate resource interactions in maize-based production systems and evaluate management strategies that alleviate above- and below-ground resource use competition while maintaining system productivity. We hypothesized that the WaNuLCAS model can accurately reproduce the spatial and temporal dynamics of resource use and yield performance in maize-based cropping systems under varying abiotic stress conditions induced by different management treatments, as observed under field conditions, thereby supporting the identification of stress-mitigating management strategies for sustainable crop production.

The study objectives were (i) to quantify patterns of resource use in contrasting upland production systems under field conditions, (ii) to determine the primary causes of yield variation associated with resource limitations, and (iii) to assess management strategies capable of improving maize performance and long-term sustainability in diversified agricultural production systems.

## 2 Materials and methods

### 2.1 Description of experimental site

The calibration and evaluation data for the WaNuLCAS model were derived from field experiments conducted in Ban Bo Wi village (13°28′ N, 99°15′ E), Ratchaburi Province, western Thailand. The region experiences a monsoonal climate with an annual rainfall of approximately 1,150 mm to 1,300 mm and mean temperatures ranging between 28°C and 29°C (max. temp. 38°C–39°C, min temp. 16°C–17 °C).Average daily incoming solar radiations are around 14 MJ m^−^² day^−^¹–16 MJ m^−^² day^−^¹. The soils of the experimental areas are shallow in nature, predominantly endoleptic Alisols and hyperskeletic Leptosols, characterized by low structural stability, stoniness, and high vulnerability to surface erosion (FAO/ISRIC/ISSS, 1998). The landscape consists mainly of steep to moderately sloping uplands, where maize (*Zea mays* L.), cassava (*Manihot esculenta*), and chili (*Capsicum annuum*) are extensively grown by smallholder farmers. Maize is typically planted following the onset of the rainy season in June, with harvest occurring between late September and mid-October. Soil physicochemical properties, including particle size distribution (texture), bulk density, pH, soil organic carbon, and cation exchange capacity, were analyzed prior to WaNuLCAS model calibration and evaluation (**Table 1**). The average values of total soil nitrogen and extractable phosphorus were 1.6 g kg^−1^ and 12.5 mg kg^−1^, respectively.

**Table 1:** Soil characteristics of experimental area used for model calibration and evaluation.

Parameter	Soil depths (cm)											
	0–5	5–15	15–30	30–45	0–5	5–15	15–30	30–45	0–5	5–15	15–30	30–45
	T1				T4				T6			
Sand (%)	50	46	54	54	42	49	52	52	42	43	50	50
Silt (%)	34	36	22	22	40	33	33	33	40	39	31	31
Clay (%)	16	18	24	24	18	18	15	15	18	18	19	19
Organic carbon (%)	1.26	1.18	0.90	0.90	1.34	1.22	0.88	0.88	1.22	1.35	1.00	1.00
Bulk density (g cm^−3^)	1.65	1.60	1.77	1.77	1.64	1.61	1.76	1.76	1.65	1.59	1.77	1.77
Soil pH	6.10	6.00	5.70	5.70	5.70	5.70	5.70	5.70	5.80	5.90	5.70	5.70
Saturated cond. (cm d^−1^)	17	16	07	07	20	19	08	08	13	17	08	08

### 2.2 Description of field experiments and data collection

Field experiments were conducted using a randomized complete block design (RCBD) having six treatments and three replicates on a hill with a 20%–25% slope gradient. The treatments were: T1: Sole maize cultivation under conventional tillage with standard fertilizer application (representing the common farmer practice); T2: Maize chili intercropping system managed with conventional tillage and fertilizer application; T3: Maize chili intercropping combined with minimum tillage and relay cropping of jack bean (*Canavalia ensiformis*), including fertilizer application; T4: Same management as T3, but with *Leucaena leucocephala* hedgerow strips established along the contour; T5: Similar to T3 (maize–chili with minimum tillage and jack bean relay), but carried out without fertilizer input; T6: Same as T4 (maize–chili with minimum tillage, jack bean relay cropping, and leucaena hedgerows), but without fertilizer application. The plot size was 13 m × 4 m. Three contrasting treatments, T1, T4, and T6, field-measured data were used as input data from WaNuLCAS calibration, while T2, T3, and T5 field-measured data were used as independent data sets for model evaluation.

Tillage operations were performed by hand-hoeing to a depth of 20 cm only in the tillage treatments, while in the treatments designated for minimum tillage, planting and weeding were carried out manually to minimize soil disturbance. Maize was manually sown at the end of June. In the intercropping treatments, one-month-old chili seedlings were transplanted on the same day. The Leucaena hedges were established two years earlier than the field experiments. In treatment T1, 17 maize rows were planted with a row spacing of 75 cm and plant-to-plant distance of 25 cm. T2, T3, and T5 contained eight maize rows with a 75 cm row-to-row distance and six chili rows with a 100 cm row-to-row distance. In the intercropping treatments, maize and chilies were planted in a sequence of two maize rows followed by two chili rows within each plot. The distance between the maize and chili rows was 100 cm. In T4 and T6, eight maize rows and four chili rows were planted at the same distance as indicated in intercropping treatments with three hedgerows, each 1 m wide, were established at the top, middle, and bottom of the plots with a 25 cm distance between the maize rows and the hedgerows. Nitrogen fertilizer in the form of urea was applied at a total rate of 62 kg ha^-^¹, split into two equal doses at 30 and 60 days after planting (DAP) maize. Phosphorus (as triple super phosphate) and potassium (as potassium chloride) were applied at rates of 11 kg ha^-^¹ and 36 kg ha^-^¹, respectively, at 30 DAP for maize. For chili, a basal application of N (urea) at 92 kg ha^-^¹ was applied at transplanting, followed by a top dressing of 92 kg ha^-^¹ N one month later, according to local recommendations.

The Leucaena hedges were pruned four times at 7, 30, 60, and 105 days after planting maize to keep the hedges at a height of 50 cm so that they may remain shorter than the maize plants to avoid the shading effect. All plots were regularly hand weeded. In the minimum tillage treatments, jack beans were planted between all maize rows one month prior to the maize harvest. These Jack beans naturally senesced during the dry season, and their residues were used as soil mulch. In all treatments, maize stalks were chopped, and in the hedgerow treatments, the pruning material was chopped and evenly spread on the soil surface within the respective plots. The amounts of Leucaena residues were 2.5 kg m^-^² and 2.2 kg m^-^² for T4 and T6 during the first experimental year, and 3.5 kg m^-^² and 3.0 kg m^-^² for T4 and T6 in the second experimental year, respectively and were applied to the respective plots to increase organic matter. Maize was harvested row-by-row for each treatment. The plants were separated into leaves, stems, and cobs, dried, weighed, and used to calculate aboveground biomass (AGB). The yields from individual rows were converted into kg per meter square to ensure comparability with the output from the WaNuLCAS model. The nitrogen and phosphorus concentrations in maize grains were determined for each row using the dry combustion method coupled with a mass spectrometer for N and inductively coupled plasma optical emission spectrometry for P measurements (Euro Elemental analyzer coupled to a Finnigan Delta IRMS, Thermo Scientific, Germany). The volumetric water content was measured using TDR probes installed at a soil depth of 0 cm–20 cm using the “Top equation” connected to a data logger. The volumetric water content was monitored throughout the maize growing season. Root length density was measured through core sampling at various points within the treatments. Root samples were collected from 0 cm–5 cm and 5 cm–20 cm soil depths.

### 2.3 WaNuLCAS setup and input data

Water Nutrient and Light Capture in Agroforestry System (WaNuLCAS) is designed to represent resource-use efficiency in wide range of production systems, including agroforestry systems. It was developed using the STELLA^©^ modeling software (Isee Systems Inc., Lebanon, USA), with a particular emphasis on capturing both above- and below-ground interactions and resource utilization between the crops present in the production system. The input data required for the simulations included encompass soil parameters (e.g., texture, organic carbon, bulk density, hydraulic conductivity, N and P contents), crop library data (growth parameters such as vegetative and generative periods, LAI), management details (planting dates, fertilizer amounts and timing, organic material applications, pruning schedules), and daily weather data (soil temperature, rainfall, evapotranspiration).

**Figure 1:**
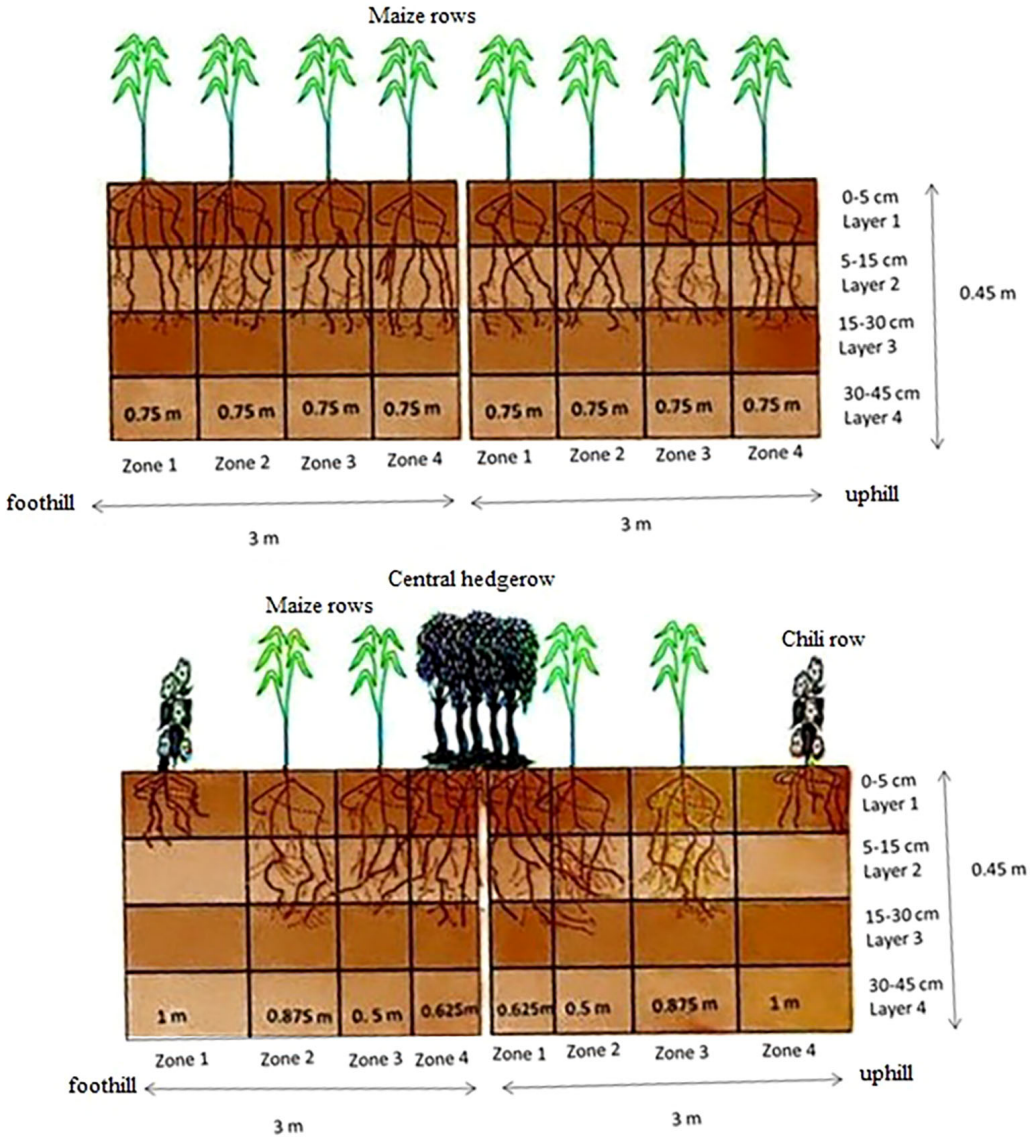
Structural setup of horizontal zones and vertical layers of the contrasting treatments in WaNuLCAS model for simulation.

The model framework represents the system horizontally through four zones and vertically through four soil layers of varying depths. For this study, three maize cropping systems from the soil conservation study were selected and divided into four horizontal zones of specific lengths representing maize, chili, or leucaena rows, and four vertical soil layers (0 cm–5 cm, 5 cm–15 cm, 15 cm–30 cm, and 30 cm–45 cm), as indicated in **Figure 1**. The soil parameters for these layers were individually entered into the pedotransfer functions (PTF) within an associated Excel file. The PTF developed by Hodnett and Tomasella (2002) was selected to generate soil hydraulic properties because it best represents tropical soil conditions. Daily rainfall and soil temperature data obtained from a weather station installed at the field site, were used as input data for the weather module of the model. WaNuLCAS simulates crop growth and development daily, and is influenced by four main factors: light, water, nitrogen, and phosphorus. The uptake of water and nutrients by plants is driven by a corresponding “demand” parameter, which is calculated as the minimum of the plant demand and potential uptake. Nutrient demand is derived from an empirical relationship between nutrient uptake and dry matter production under non-limiting conditions, accounting for luxury uptake, compensation for past deficits, and nitrogen fixation. In nutrient-deficient situations, the target N content is compared with the current nutrient content, and the deficit can be met through atmospheric fixation and nutrient addition. The WaNuLCAS Crop_PosGro parameters indicate the magnitude of the constraining factors (N, P, water, and light) for plant growth per zone, varying from 0 (no growth/high stress) to 1 (no stress). Light capture was calculated as a function of the crop leaf area index (LAI) and relative height in each zone. For trees and tall plants in the production systems, light capture is separated into components for leaves (LAI) and branches (branch area index, BAI), allowing the model to account for shading even when trees are leafless.

The water and nutrient uptake by plants is driven by the corresponding ‘demand’ parameter in WaNuLCAS model interface as:


Uptake (water, nutrient)=min (demand, potential uptake)

Whereas under nutrient deficient situations, target N content is contrasted with current nutrient content and can be met by atmospheric fixation and or by nutrient additions in the model interface. WaNuLCAS Model calculates it as:


$$CN\_Demand=CN\_Deficit*\;{\left(1-0.5*\;Cq\_stage\right)}^{2}$$

Where the *CN_Demand* is the crop nutrient demand (g m^−2^), *CN_Deficit* is crop nutrient deficit (g m^−2^), *Cq_stage* is the crop sequence stage.

In WaNuLCAS interface the total light capture in each canopy layer is calculated by Beer’s Law;


$$\begin{array}{l} {TotLightCap}_{j}=1-\sum_{k=1}^{j-1}{TotLightCap}_{k}\\ -e^{-\sum_{i}\left({kLLight_{i}*LAI_{i}-kBLight_{i}*BAI}_{i}\right)} \end{array}$$

where *TotLightCap_j_*is the total light capture (g m^−2^) by each canopy layer (*j*), *kLLight* is the light extinction coefficient for leaves, *LAI* is the leaf area index, *kBLight* is the light extinction coefficient for branches.

### 2.4 Model calibration and evaluation

The model was parameterized and calibrated using a two-year dataset from treatments T1, T4, and T6, which represented strongly contrasting management and cropping practices in this study. Independent treatments T2, T3, and T5 were used for model evaluation. For the maize sole cropping control (T1), four horizontal zones, each 0.75 m wide with a single maize row, were established for calibration, matching the planting pattern of the field trial. For the agroforestry systems, two distinct spatial situations were considered: “above hedge” and “below hedge.” In the “above hedge” configuration, hedgerows were placed in zone one, with maize in the next two zones and chili in zone four. In the “below hedge” configuration, hedges were placed in zone four. During the calibration process, various WaNuLCAS parameters were modified and applied to improve the model fit to the observed data (**Table 2**). These changes were only made during the calibration process, and the modified values fixed during the calibration process were used during the evaluation of the model.

**Table 2:** Model inbuilt parameter with their default and modified values used during model calibration process.

Model parameters with description	Default values	Modified values
Cq_HBiomConv (Factor for conversion of crop biomass increment (up to crop stage 1) to crop height)	7	1.2
Cq_KLight (Light extinction coefficient for the crop canopy = efficiency of crop foliage in absorbing light)	0.65	0.68
Cq_GroMax (Maximum daily dry matter production rate at full light capture, under local conditions)	0.014	0.07
Cq_Gseed [Seed weight (initial C_Carb Hydr Reserves to be used for growth)]	0.004	0.03
Cq_MaxRemob (Maximum proportion of stem and leaves remobilized per day to the Carb Hydr Reserves pool, from which it can be used for growth of storage component)	0.05	0.02
Cq_RainWStorCap (Rainfall water stored as thin film at leaf surface)	1	0
Cq_MycMaxInf (Fraction of crop roots infected by mycorrhiza for a soil layer where the Rt_MTInfFrac parameter is 1)	0.25	0.15
Cq_RelLUE_stage 0 (Crop relative light use efficiency at stage zero)	1.72	0
Cq_RelLUE_stage 1 (Crop relative light use efficiency at stage 1)	1.02	0.2
RtCLrvm_1 (Maximum crop root length density in 1st soil layer; corresponds to Rt_ACType = 0 and Cq_AType)	5	7
RtCLrvm_2 (Maximum crop root length density in 2nd soil layer; corresponds to Rt_ACType = 0 and Cq_AType)	3	5
RtCLrvm_3 (Maximum crop root length density in 3rd soil layer; corresponds to Rt_ACType = 0 and Cq_AType)	0.3	0.9

### 2.5 Resource use efficiency scenarios

Successful calibration and evaluation of the model followed a series of scenarios developed not only to identify the causes of above- and below-ground resource use in the studied production systems by evaluating their impact on crop production, but also to test potential crop management options to mitigate the deficient resource effects on productivity. Each scenario was simulated over a five-year runtime. Three main scenarios were developed: scenario 1 focused on light-use efficiency through pruning management, scenario 2 was organized for fertilization management strategies, and scenario 3 was water management (**Table 3**).

**Table 3:** Scenario analysis used for simulating the resource use management.

Scenario 1: Light/radiation management through prunings			
A	Baseline	With no. of pruning done in field experiments
B	Continuous pruning	Hedges were pruned once in a month
C	No pruning	Hedges were not pruned for five years
Scenario 2: Fertilization management			
a	Baseline	Farmer practice (62N:11P kg ha^−1^)
b	Double N/standard P	124N:11P kg ha^−1^
c	Standard N/double P	62N:22P kg ha^−1^
d	Double N/double P	124N:22P kg ha^−1^
Scenario 3: Water management			
a	Baseline	Simulations with rainfall during experiments
b	Irrigation	Irrigation was applied on dry periods during growing season (10 mm water per dry day)

### 2.6 Statistical analysis

To assess model performance, the goodness of fit (GOF) procedure suggested by Loague and Green (1991) was used to compare the observed and simulated aboveground biomass of maize. The mathematical expressions are:

Modeling efficiency (EF);


$$\text{EF}=\left(\sum_{i=1}^{n}{\left(Oi-\bar{O}\right)}^{2}-\sum_{i=1}^{n}{\left(Pi-Oi\right)}^{2}\right)/\sum_{i=1}^{n}{\left(Oi-\bar{O}\right)}^{2}$$

Root mean square error (RMSE);


$$\text{RMSE=}{\left[\sum_{i=1}^{n}{\left(Pi-\bar{O}\right)}^{2}/n\right]}^{0.5}.\frac{100}{\bar{O}}$$

where *O_i_* are the observed values, *P_i_* are the predicted values, *n* is the number of observations or samples and *Ō* is the mean of observed values. RMSE values are in kg m^-2^.

Square of the Pearson correlation coefficient (R2);


$$R^{2}=1-\frac{SSres}{SSres}$$

where *SSres* is the sum of squares of the residual errors (the difference between observed and simulated values), and *SStot* is the total sum of squares (the difference between observed values and their mean, squared).

For a good performance of model is better to get the values of EF, RMSE and R^2^ as close as possible to 1, 0, and 1, respectively.

## 3 Results

### 3.1 Model calibration and evaluation

**Figure 2:**
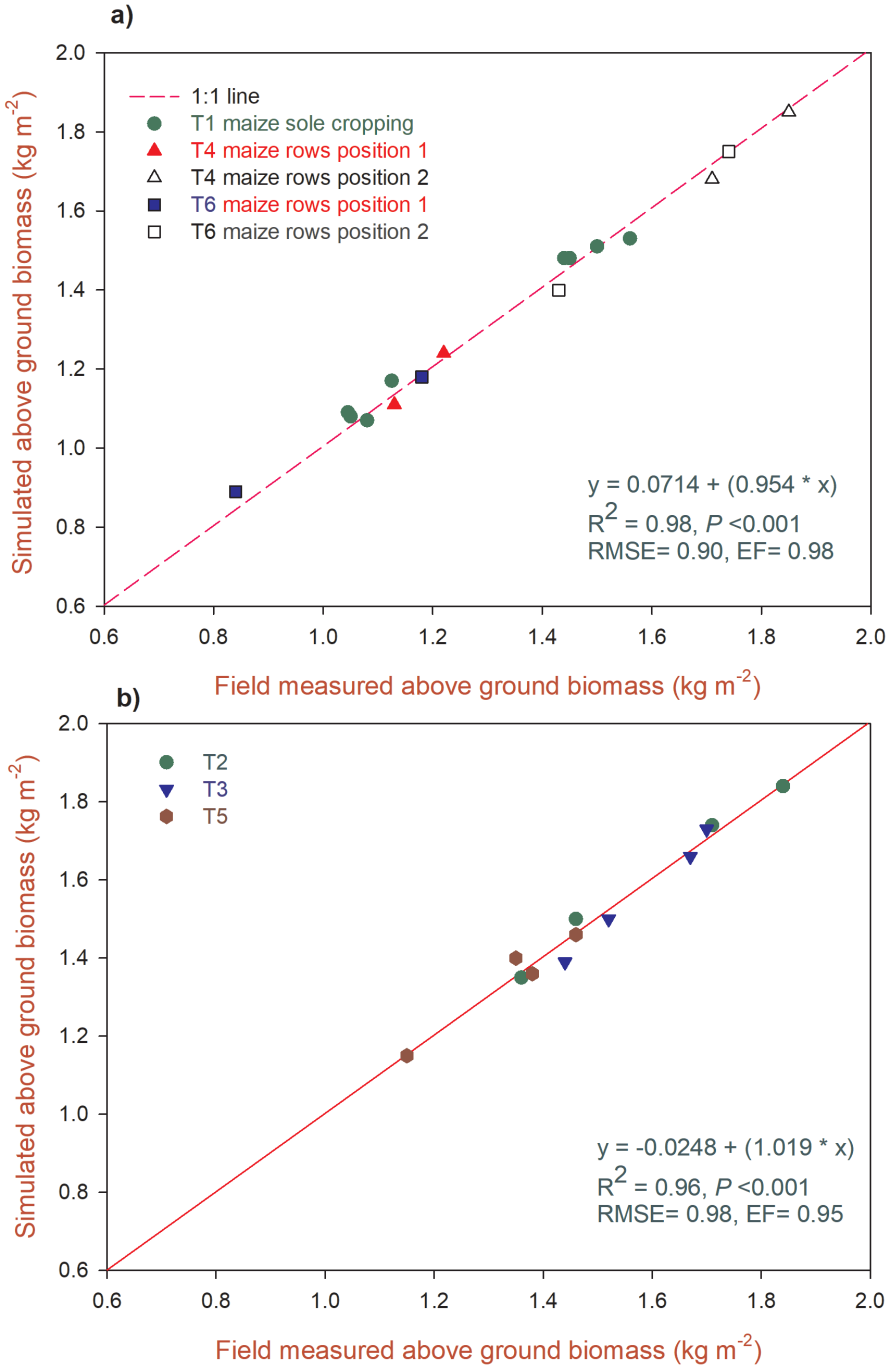
Relationship between field measured and simulated maize above ground biomass observed during **(a)** model calibration and **(b)** evaluation with regression and statistical analysis.

The model was calibrated with maize sole cropping (T1), maize–chili intercropping with hedges and fertilizer application (T4), and maize–chili intercropping with hedges and without fertilizer application (T6) treatments row yields. The relationship between field-measured and simulated aboveground biomass (kg m^-^²) is shown in (**Figure 2A**) the model calibration dataset and (**Figure 2B**) the model evaluation. The dotted line represents a 1:1 line. In **Figure 2A**, the data points correspond to the treatments used to parameterize and tune the model (T1: maize sole cropping; T4 and T6: maize in specific row positions within an intercrop system with and without fertilizer application). The fitted regression line (y = 0.0714 + 0.954x) was very close to the 1:1 line, indicating minimal overall bias. The high coefficient of determination (R² = 0.98), highly significant p-value (p <0.001), low Root Mean Square Error (RMSE = 0.90 kg m^-^²), and high Modeling Efficiency (EF = 0.98) collectively demonstrate an excellent model fit for the calibration dataset. The model successfully captured the variability and magnitude of the measured biomass for these treatments.

**Figure 2B** shows the independent evaluation/validation of the calibrated model using a separate dataset that was not used during the calibration process (Treatments T2, T3, and T5). This is most critical test for the predictive capability and robustness of the model. The regression line (y = −0.0248 + 1.019x) between the observed and simulated data was very close to the 1:1 line. The performance statistics were slightly lower than the calibration results but still showed great acceptability (R² = 0.96, P <0.001, RMSE = 0.98 kg m^-^², EF = 0.95). This indicates that the model not only fits the data on which it was trained but also performs reliably when predicting new, independent data with a high degree of accuracy and minimal error.

### 3.2 Simulation of production system performance

**Figure 3:**
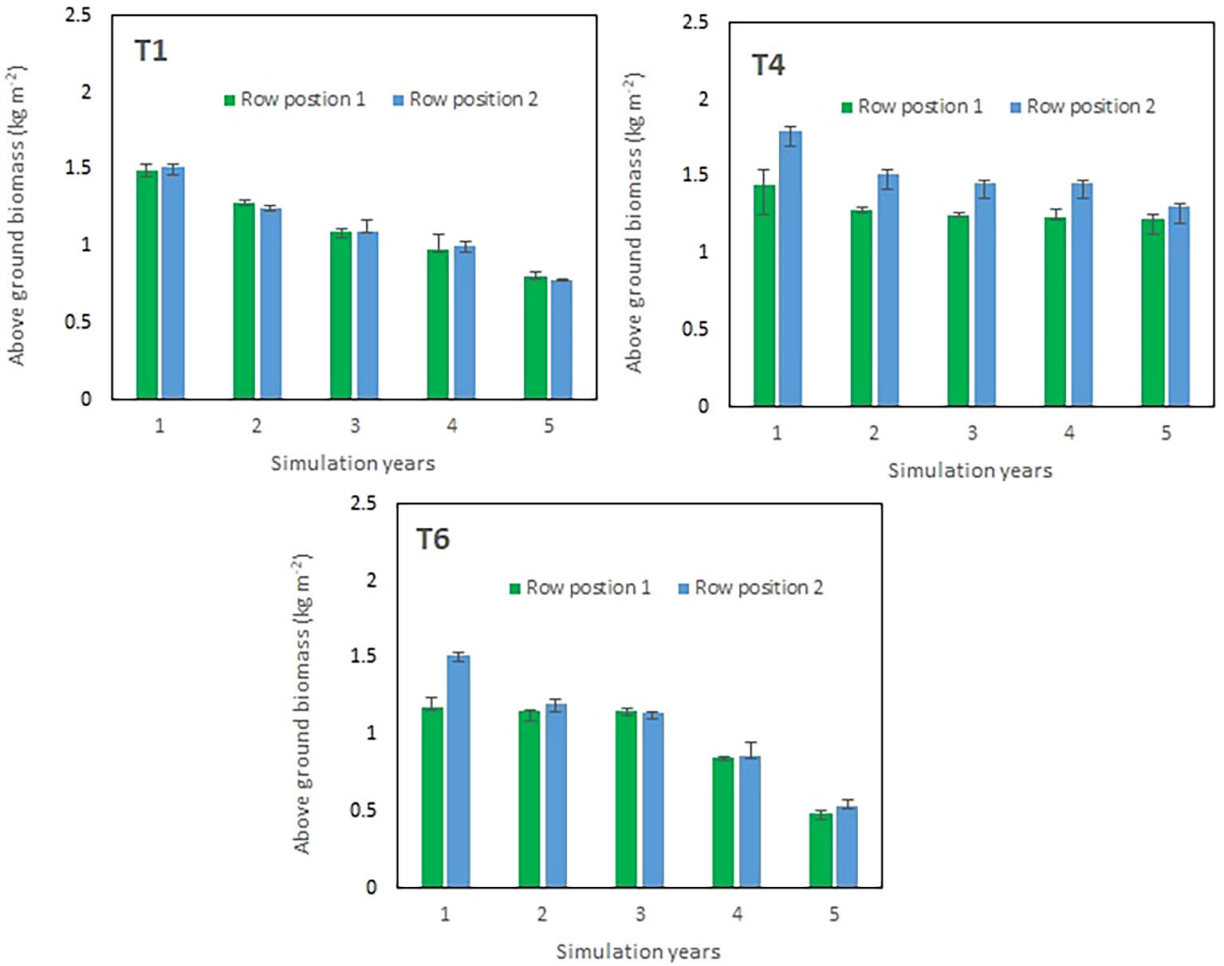
Simulation of production system performance over five consecutive years at two distinct maize row planting positions i.e row position 1 (maize row planted next to hedgerows), row position 2 (maize row planted away from hedgerows) in hedgerows containing treatments (T4 & T6). Vertical bars indicate the standard error.

The five-year simulation of aboveground biomass revealed distinct temporal patterns among treatments and row positions, especially in the intercropped treatments (**Figure 3**). In general, all treatments exhibited a gradual decline in biomass over time, suggesting a cumulative reduction in productivity over the simulation years. In the maize sole cropping treatment (T1), the aboveground biomass was highest in the first year (≈1.5 kg m^-^²) for both corresponding row positions on the slope and declined progressively thereafter, reaching approximately 0.9 kg m^-^² by year 5. The differences between the row positions were negligible throughout the simulation period, indicating a uniform response of crop growth within this treatment. In maize–chili intercropping with hedges and fertilizer application (T4), the initial biomass was higher than that in T1, particularly at row position 2, which was away from the hedgerows, which achieved approximately 1.8 kg m^-^² in the first year. All production systems gradually decreased biomass in subsequent years, but row position 2 consistently maintained slightly greater productivity than row position 1 (planted close to hedges), suggesting potential spatial or microenvironmental advantages influencing crop performance, especially in T4 and T6. By the fifth year, biomass values converged to the lowest across both positions in all treatments. Maize–chili intercropping with hedges and without fertilizer application (T6) produced initial biomass corresponding to T1, with row position 2 reaching nearly 1.5 kg m^-^² in year 1. However, this treatment showed the steepest decline in productivity, with biomass decreasing to below 0.8 kg m^-^² by year 5. Despite its early performance, the faster rate of decline implies limited sustainability of biomass production under this treatment in the long term.

The decreasing trend in simulated biomass across all treatments likely reflects the cumulative effects of soil nutrient depletion, reduced soil moisture availability, and other stress factors represented within the model. The consistent superiority of row position 2 in T4 and T6 further highlights the role of spatial variability in determining crop growth performance under different management conditions.

### 3.3 Simulation of resources use in various production systems

**Figure 4:**
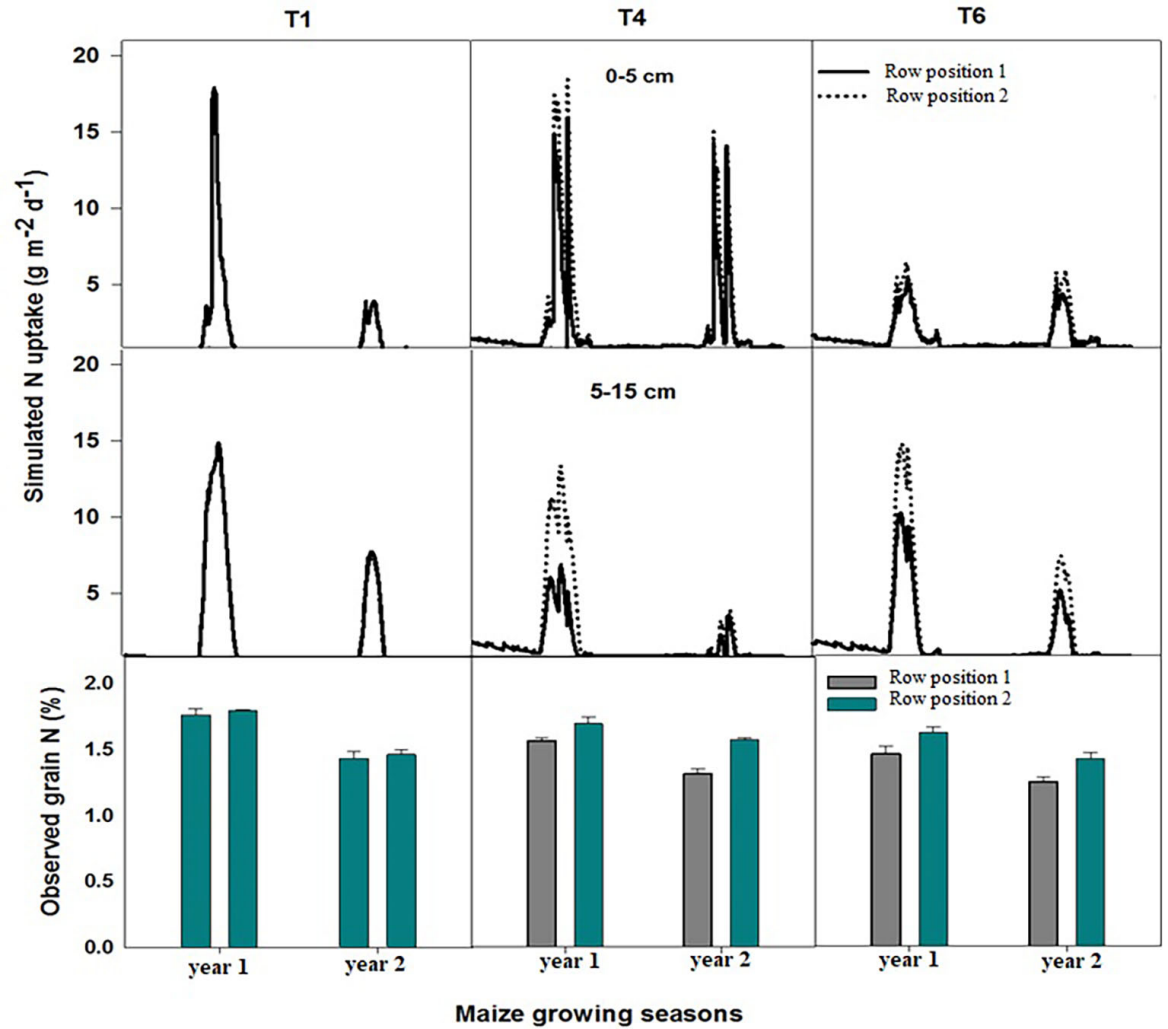
Simulated nitrogen (N) uptake over two consecutive years planting in T1, T4 and T6 at 0-5 and 5-15 cm soil depth along with nitrogen content of the grains, measured during two-year field trials. In T4 & T6, row position 1 representing maize row planted next to hedgerows while row position 2 is the maize row planted away from hedgerows.

Simulated nitrogen uptake exhibited distinct patterns among the treatments and row positions, reflecting the combined effects of management and spatial variation (**Figure 4**). Across all treatments, nitrogen uptake showed moderate interannual variability, whereas the observed grain nitrogen content displayed a corresponding trend consistent with the simulated uptake levels.

In the maize sole cropping treatment (T1), simulated N uptake remained comparable to that of the other treatments during the first year of the experiment, while it was lowest in the 2nd year simulation period. The observed grain N content in both rows was lower than that of the other treatments in both years. Row performance was similar in terms of nitrogen uptake and N content in grains, suggesting similar N assimilation and translocation to grains under this treatment in both years at both depths (0 cm–5 cm and 5 cm–15 cm). First-year uptake of nitrogen was higher than the 2nd year with similar trends in grain nitrogen concentration. The differences between the row positions were minimal, indicating uniform crop N acquisition under the prevailing conditions. In maize–chili intercropping with hedges and fertilizer application (T4), simulated N uptake increased notably relative to T1 in the initial years, followed by a slight decline toward the 2nd year of simulation. The corresponding observed grain N content was higher, particularly in row position 2, which consistently outperformed row 1, which was close to the hedges. This alignment between the simulated uptake and measured grain N concentration indicates a realistic model representation of nitrogen dynamics under this treatment. In T4, the simulated nitrogen uptake was higher at 0 cm–5 cm as compared to 5 cm–15 cm depth in both years with similar trends of uptake at specific row positions.

The model simulated the lowest nitrogen uptake by maize plants at both positions in the maize–chili intercropping with hedges and without fertilizer application treatment (T6) at 0 cm–5 cm soil depth during both studied years as compared to T1 and T4, while the model simulated highest N uptake values at 5 cm–15 cm soil depth in the first-year simulation, but a marked decline was evident in later year simulations. The nitrogen uptake was higher in row position 2 than in row position 1 at both soil depths. The observed grain N content followed a similar pattern, with initially higher values that gradually decreased over time. The simulated N uptake and grain nitrogen concentration suggest that T6 may have contributed to reduced N-use efficiency or soil N depletion over successive years owing to the unavailability of nitrogen.

Furthermore, simulated and observed data showed consistent temporal and spatial trends as observed under field conditions, supporting the model’s reliability in capturing treatment-dependent variations in nitrogen uptake and its partitioning. The higher simulated uptake and grain N in row position 2 across T4 and T6 highlight the potential role of microenvironmental heterogeneity probably due to diversified cropping, in influencing nitrogen acquisition and utilization efficiency.

**Figure 5:**
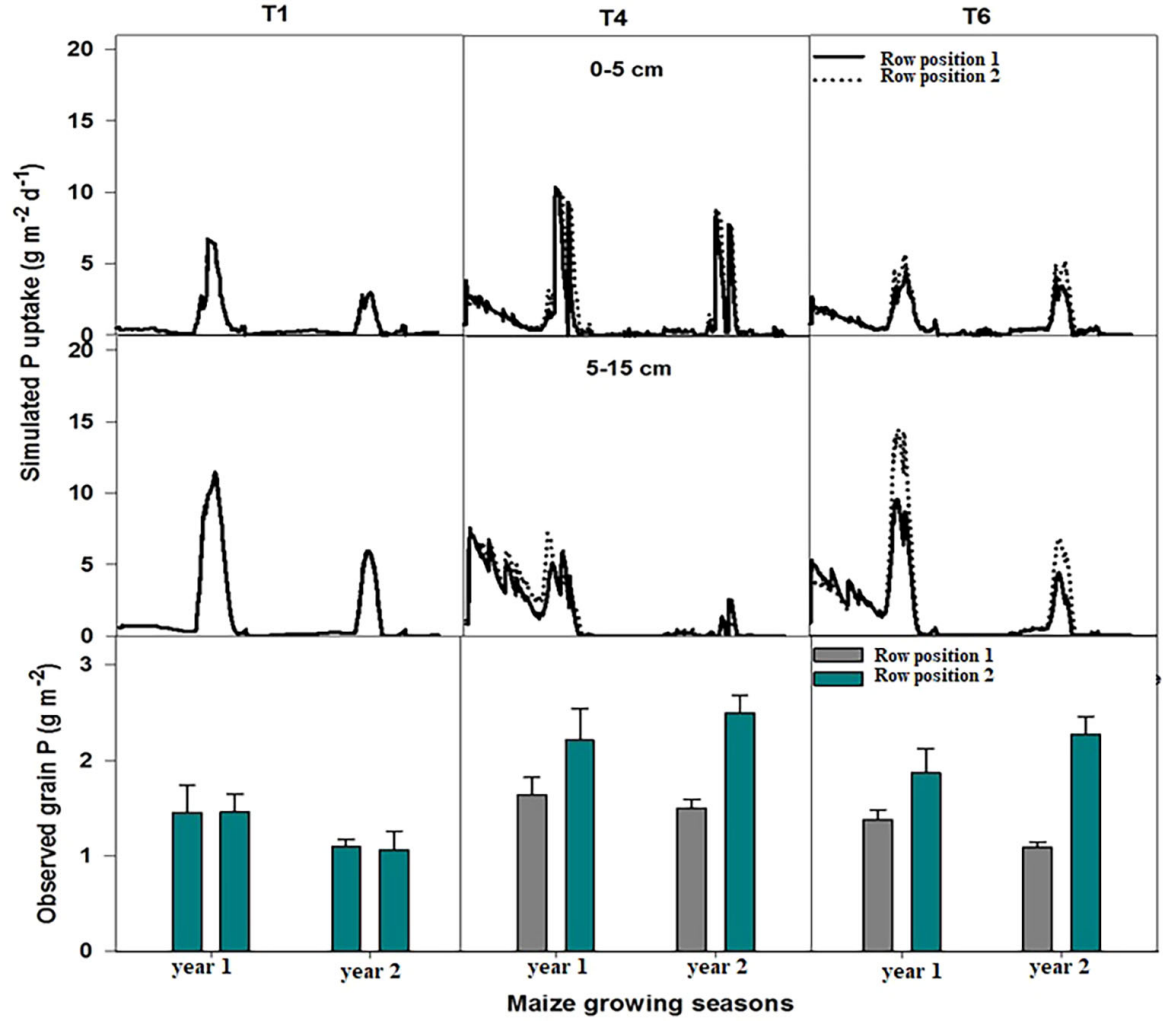
Simulated phosphorous (P) uptake over two consecutive years planting in T1, T4 and T6 at 0-5 and 5-15 cm soil depth along with phosphorous content of the grains, measured during two-year field trials. In T4 & T6, row position 1 representing maize row planted next to hedgerows while row position 2 is the maize row planted away from hedgerows.

The simulated phosphorus (P) uptake across soil depths and treatments also revealed clear differences in both uptake magnitude and depth distribution, reflecting the corresponding observed grain P content (**Figure 5**). Simulated P uptake was greater at the surface layer (0 cm–5 cm) except T6 compared with the subsurface layer (5 cm–15 cm). At the 0 cm–5 cm soil depth, simulated P uptake in T1 remained relatively low across both simulation years, indicating the limited availability of labile phosphorus in the surface layer. In T4, P uptake usually increased, but lower simulated P uptake was indicated, particularly in maize planted at row position 1. These results suggest improved management practices in T1 and T4, which enhanced surface P acquisition.

At the 5 cm–15 cm depth, simulated P-uptake values were lower in T4, but the same trend persisted. In T1, simulated P uptake increased in the first simulated year, whereas T4 simulated P uptake declined in both years, and T6 simulated P uptake also increased compared to 0 cm–5 cm soil depth and T4 at 5 cm–15 cm depth.

The observed grain P content exhibited a similar treatment-dependent pattern, validating the simulation trends. Mean grain P concentrations were lowest under T1, highest under T4, and moderate under T6 in both years. The higher grain P content in row position 2 of T4 and T6 indicates improved translocation efficiency or localized soil fertility differences. The close agreement between the simulated P uptake and observed grain P concentration across treatments and depths suggests that the model realistically represents phosphorus acquisition and partitioning dynamics under the tested management conditions.

Moreover, the simulated light captured by the canopy showed no differences in light use efficiency in all correspondence rows in T1, T4, and T6, indicating no light stress in the studied years. Similarly, the model showed no water stress indication in all the treatments, as water was applied during the dry period in the field experiment. The “Crop_PosGro” model parameters indicated a magnitude of 1 (no stress) in the case of water and light capture, indicating that both water and light are non-constraining factors in the studied production systems.

### 3.4 Grain δ¹³C variability across treatments and row positions

**Figure 6:**
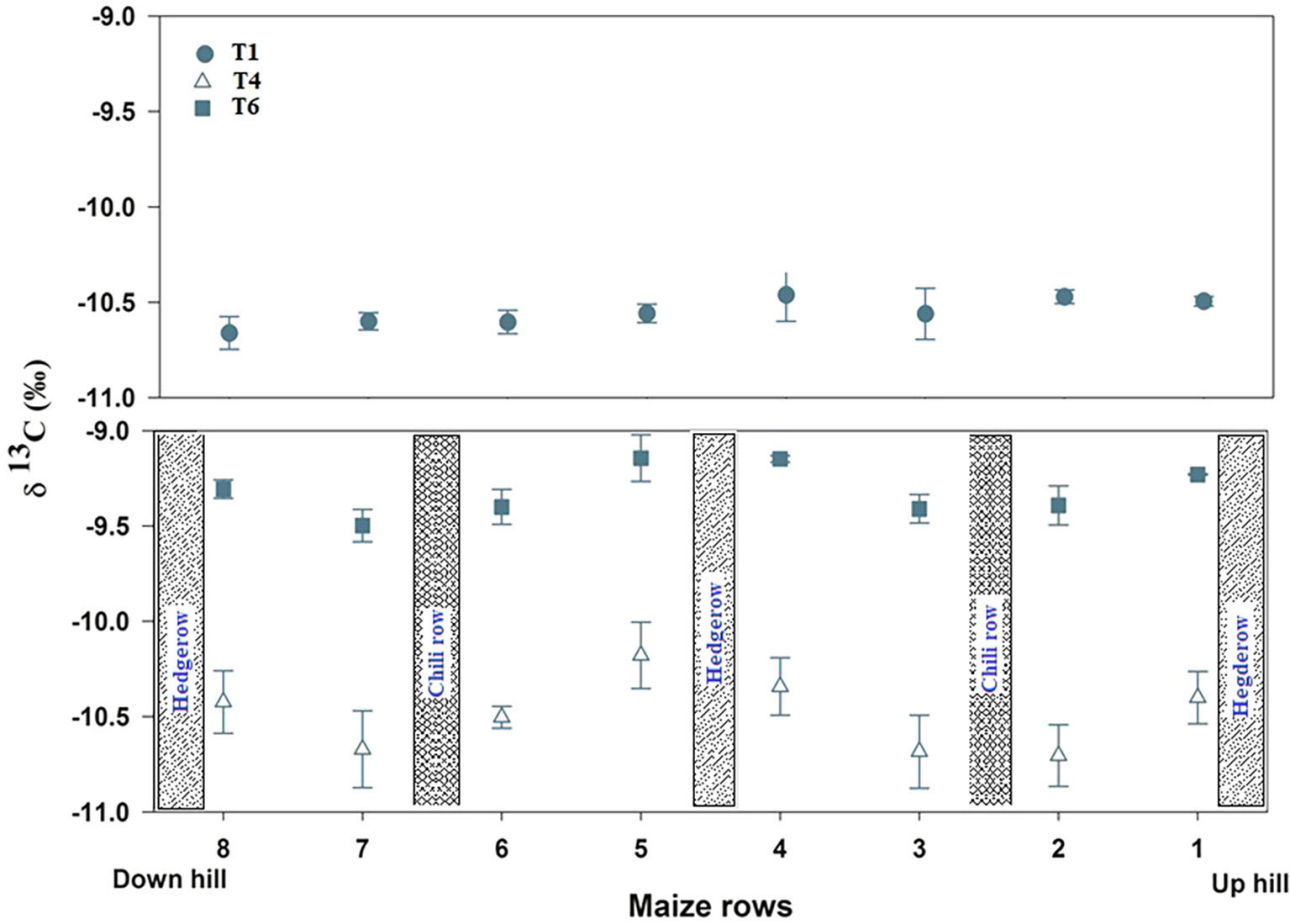
Spatial trend of grain delta 13C in T1, T4 and T6 in eight maize rows. The data presented here is the average of two years field experiments. Vertical bars indicate the standard error.

Grain δ¹³C values varied among treatments and row positions, indicating differences in plant water-use efficiency and microenvironmental influence across the cropping configurations (**Figure 6**). In the sole maize system (T1), δ¹³C values were relatively uniform across all eight rows, ranging from −10.47‰ to −10.66‰. The limited variation suggests that in the absence of hedgerows and intercropping, maize plants experienced comparatively homogeneous soil moisture and evaporative demand conditions across the plot. In the maize–chili + hedgerow system with fertilizer (T4), δ¹³C values were more spatially differentiated. The four maize rows positioned closer to the hedgerows had less negative δ¹³C values (−10.40, −10.34, −10.18, −10.42‰), indicating higher water availability, likely due to moderate shading, reduced evaporative load, and improved microclimatic moisture retention near the hedgerows. In contrast, the four maize rows positioned away from the hedgerows had more negative δ¹³C values (−10.70, −10.68, −10.50, −10.67‰), suggesting water or nutrient stress. The spatial gradient in T4 also demonstrated that hedgerow proximity modified resource use, even under fertilized conditions.

In the maize–chili hedgerow system without fertilizer application (T6), the δ¹³C values were less negative than those in T1 and T4. Maize rows located closer to hedgerows showed the highest δ¹³C values (−9.26, −9.15, −9.14, −9.31‰), suggesting strong stomatal regulation and efficient carbon assimilation under moderated microclimatic influences, while maize rows located away from hedgerows (row position 2) showed slightly more negative values (−9.39, −9.41, −9.40, −9.50‰) than those of maize rows positioned close to the hedgerows in T6.

### 3.5 Soil volumetric water content

**Figure 7:**
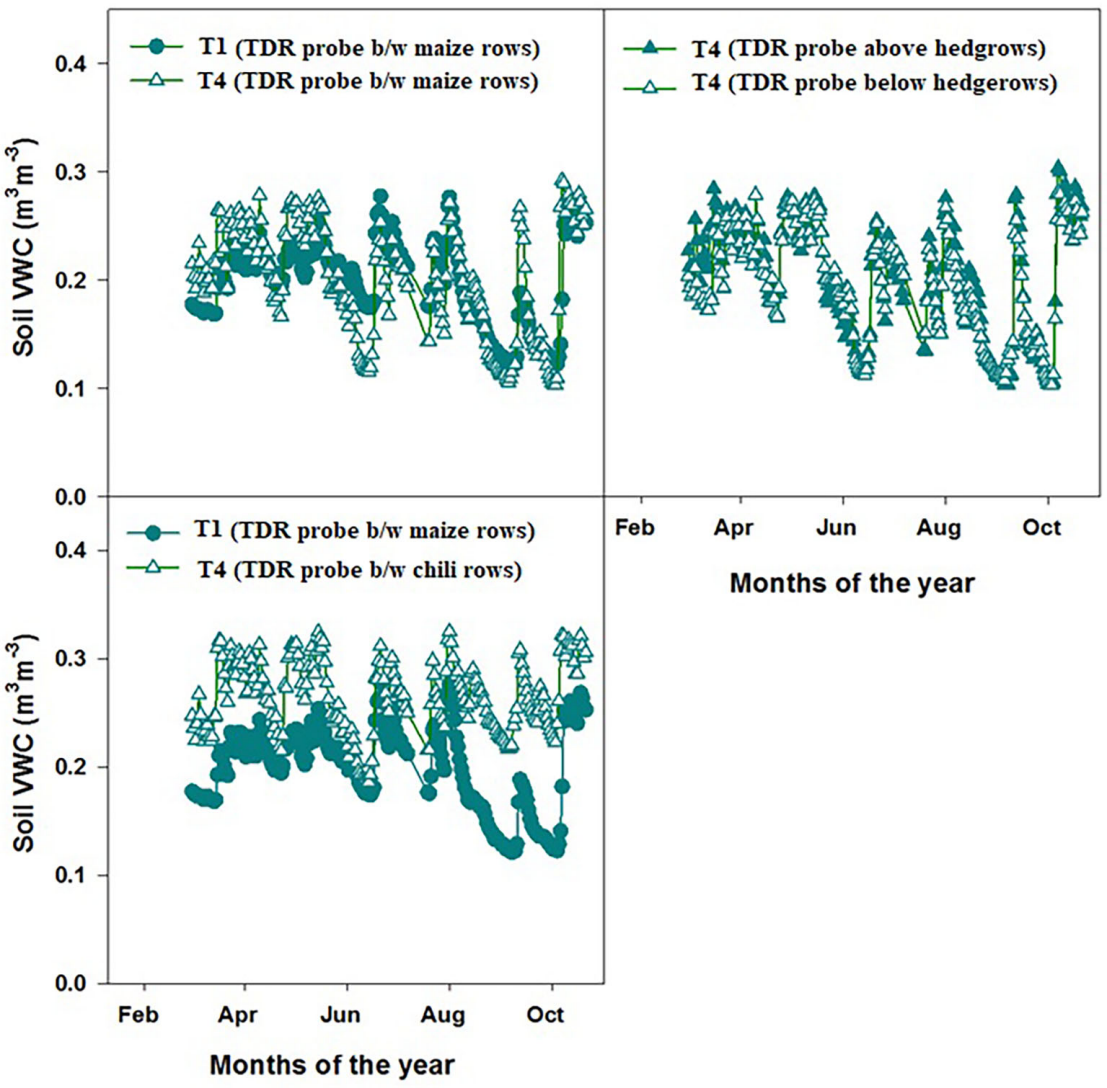
Soil volumetric water contents measured through time domain reflectometry (TDR) in T1 and T4 during the growing period. Probes were installed at 0 cm–20 cm depth. Data is the average of two growing seasons.

The soil volumetric water content (VWC) trends observed in the sole maize system (T1) and the maize–chili intercropping system with hedgerows and fertilizer application (T4) differed noticeably at 0 cm–15 cm soil depth (**Figure 7**). When soil moisture was measured between maize rows in both treatments, T4 consistently maintained a higher VWC than T1, indicating improved soil water retention under the hedgerow-based system.

Similar trends in soil VWC were observed between maize rows in T1 and between chili rows. Despite the fact that chili plants have a smaller canopy than maize, the soil in T4 exhibited higher moisture levels.

The soil VWC above and below the central hedgerow in T4 showed small differences in the soil moisture gradient at both positions. The area closer to the hedgerow (upslope side) retained slightly more moisture than that further downslopes. This pattern is consistent with localized shade effects, organic matter deposition from periodic pruning, and improved soil structure in the hedge root zone. However, the observed gradient did not indicate severe competition for water depletion from hedgerow root activity. These results demonstrate that the integration of hedgerows in T4 improved soil water conservation across the crop rows compared to sole maize cultivation in T1.

### 3.6 Root length density distribution

**Figure 8:**
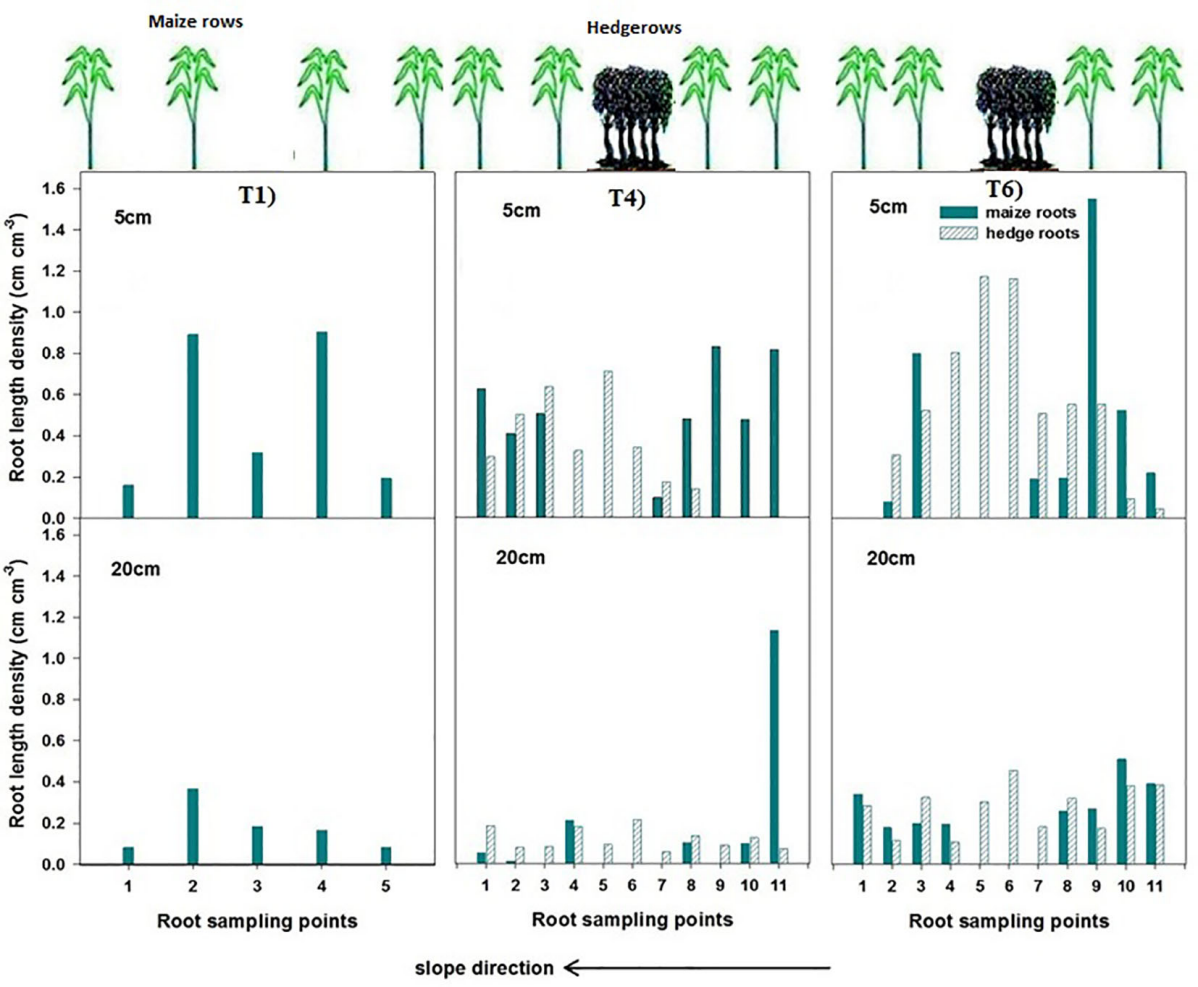
Root length density (RLD) of maize and hedgerows in T1, T4, and T6. The samples were collected at the time of maturity.

Root length density patterns measured during field experiments differed distinctly between the treatments and positions relative to hedgerows and maize rows (**Figure 8**). In the sole maize system (T1), RLD was uniformly distributed across the maize rows at similar slope positions as those of maize rows in T4 and T6, showing minor variation along the slope within T1 conditions, indicating even availability of rooting space under sole cropping conditions. However, T4 (maize–chili intercropping with hedgerows and fertilizer application) showed a clear spatial gradient in maize RLD at various positions. The maize rows located adjacent to the hedgerow (row position 1) exhibited noticeably lower RLD than rows positioned further away from the hedge (row position 2). In contrast, Leucaena displayed a high RLD at the hedgerow zone, with roots extending laterally into the maize root zone. This overlap of maize roots and hedgerows suggests below-ground interaction of both components of the production systems for nutrients and water.

In maize–chili intercropping with hedges and without fertilizer application (T6), both maize and hedgerows produced higher RLD than in T1 and T4 at both soil depths. The RLD of maize and hedgerows at all sampled positions increased at both soil depths, with dominant root overlap at 0 cm–20 cm soil layer. This confirms that the absence of fertilization intensified RLD, likely due to a greater reliance on soil nutrient pools. The lack of nutrient availability directed the roots to penetrate deeply for nutrient search for sustainable growth.

### 3.7 Simulation of plant resource uses management

**Figure 9:**
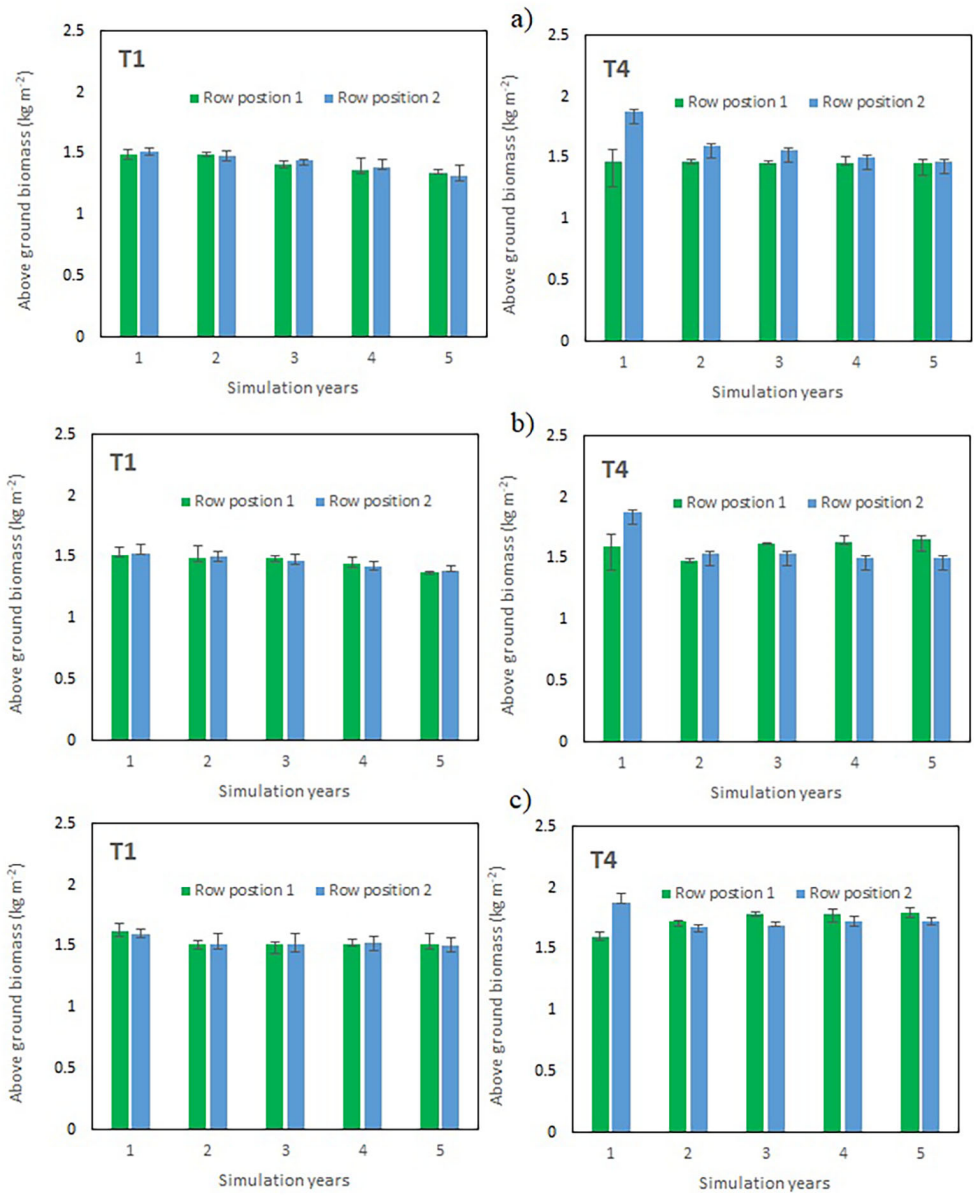
Sustainable resource use management over five growing seasons simulations in T1 and T4 by the application of fertilizers at the rate of a) Double N/ standard P (124N:11P kg ha-1), b) Standard N/ double P (62N:22P kg ha-1), c) Double N/ double P (124N:22P kg ha-1). Vertical bars indicate the standard error.

The model simulations demonstrated distinct responses of aboveground biomass to changes in nitrogen (N) and phosphorus (P) supply in both the sole maize system (T1) and the maize–chili intercropping with hedgerows and fertilizer application (T4). These responses were consistent across five consecutive simulation years and revealed key differences in system resilience between sole cropping and hedgerow-based intercropping (**Figure 9**).

When nitrogen (N) input was doubled while maintaining the standard phosphorus (P) rate (120N:11P), biomass production remained relatively stable across years in both T1 and T4 (**Figure 8A**). However, the magnitude and spatial distribution of the biomass differed between systems. In T1, the biomass remained largely similar between Row Positions 1 and 2, indicating a uniform response to added N under sole maize. In contrast, T4 exhibited consistently higher biomass in Row Position 2 (located further away from the hedgerow) than in Row Position 1 during the early simulation years. This pattern aligns with the known belowground competition effects between hedgerow roots and adjacent maize rows. Over time, the biomass difference between row positions in T4 diminished, suggesting that the increased N availability partially mitigated the competitive stress near the hedgerow. Overall, doubling N supported yield maintenance in both systems, but spatial variability persisted in T4. In the second scenario, P application was doubled, keeping the nitrogen application standard as used under field conditions (62N:22P), which also sustained maize biomass over time, particularly in T4 (**Figure 8B**). In T1, the biomass declined slightly but remained stable across row positions. However, in T4, the biomass in Row Position 1 substantially increased compared to that in Row Position 2 from the third simulation year. These results highlight that P addition alone alleviated hedgerow-induced competition by increasing the biomass yield of maize rows planted close to the hedges (maize row position 1).

Simultaneously increasing both N and P (120N:22P) resulted in the most favorable and stable biomass trends across the five-year simulation period (**Figure 8C**). In both T1 and T4, biomass levels were higher and less variable than in scenarios (a) and (b). Notably, in T4, the difference between Row Positions 1 and 2 diminished considerably, indicating that a sufficient nutrient supply reduced the competitive disadvantage associated with hedgerow proximity. This combined NP-input scenario effectively enhanced overall productivity and improved temporal sustainability, suggesting that nutrient limitations and competition effects can be jointly alleviated when both major macronutrients are balanced in the environment.

## 4 Discussion

The combined field measurements and WaNuLCAS simulations in the current study enhanced our understanding of the prevailing resource-use trends under diversified planting conditions. Furthermore, the results indicated that nutrient competition (N and P) was the dominant interaction affecting maize performance planted close to the hedgerows, whereas water and light were not limiting resources in the studied production systems. The soil, weather, and management conditions, except for the treatments, were uniform at the field site, which strengthened the treatment effects, as observed under field conditions. The soil properties across T1, T4, and T6 were generally similar, with all plots showing a sandy loam texture in the upper layers and a slightly higher clay content at 15 cm–30 cm. Organic carbon declined with depth in all treatments, but T4 showed slightly higher topsoil OC, suggesting modest improvement under hedgerow-based management (hedges were planted two years before the experimental years for their establishment). The bulk density increased with depth. The soil pH was consistently moderately acidic (5.7–6.1) across all treatments. The saturated hydraulic conductivity decreased with depth, indicating similar fertility gradients in the study area.

### 4.1 Resource use and competition management

Grain δ13C values (**Figure 6**), soil volumetric water content (VWC) (**Figure 7**), and WaNuLCAS water balance modules consistently showed that soil moisture remained above the critical threshold for maize growth across both treatments (T1 and T4). The PosGro water capture function of WaNuLCAS indicated non-limiting soil water conditions throughout the growing period. This was further supported by the absence of δ13C enrichment in maize grains, indicating no waste stress during the growing season (Hussain et al., 2015).

This aligns with previous studies showing that *L. leucocephala* typically develops a deep-rooting profile, accessing water below the maize rooting zone and thereby reducing competition for shallow moisture (Pachas, 2017; Montejo-Martínez et al., 2020). The greater VWC observed in T4 (with hedgerows) suggests that hedgerow presence contributed to microclimate modification, improved soil organic matter, and reduced evaporation, as reported in Leucaena-based systems in South Asia and East Africa (Sanchez and McCollin, 2015; Montgomery et al., 2020; Worku and Mekoya, 2025). Therefore, both field and model evidence confirm that water was not a limiting factor and that hedgerows did not induce water stress in maize in either spatial position.

Hedgerow height was actively maintained at a height of approximately 50 cm, keeping it below the maize canopy, ensuring no light limitation, especially on the canopy of maize rows planted close to the hedges. WaNuLCAS simulations (Light cap–PosGro function) demonstrated that photosynthetically active radiation (PAR) at the canopy level was not reduced in either maize row position. This is consistent with controlled-shading studies showing that light competition in alley cropping occurs primarily when woody species exceed the cereal canopy height (Ladux et al., 2024; Raskin, 2025). Thus, the negligible light competition here was a direct result of appropriate hedgerow pruning, showing that management decisions can override potential structural competition.

Field measurements showed significantly lower grain nitrogen and phosphorus concentrations in maize grown adjacent to hedgerows (row position 1) than in the rows positioned further away (row position 2) due to nutrient competition (Zhao et al., 2025), as depicted in **Figures 4**, **5**. This pattern was reflected in the WaNuLCAS simulations, which predicted higher N and P uptake in maize planted away from the hedgerows. These findings reflect the competitive nutrient uptake behavior of Leucaena, whose root network overlapped with the maize roots measured at 0 cm–20 cm soil depth, overlapping directly with maize rooting zones (Pachas, 2017). The root length density maps in this study (**Figure 8**) confirmed dense hedgerow rooting near maize position 1, indicating direct nutrient competition (Zhao et al., 2025), particularly in T4, where root overlap was highest. The rooting pattern in T6, with its higher RLD, indicated that nutrient unavailability directed the plant to grow deep into the soil for nutrient fixation (**Figure 8**).

Furthermore, the δ13C values in maize plants near the hedgerows exhibited δ13C signatures similar to those of the non-fertilized controls (T6), indicating limited nutrient-driven stomatal activity, rather than water limitation. δ13C enrichment linked to nutrient limitation rather than drought is increasingly recognized as a diagnostic marker in agroforestry systems (Hussain et al., 2015; Cernusak and Ubierna, 2022). Thus, the poor yield performance of maize adjacent to hedgerows is best explained by nutrient competition, not by water or light scarcity.

### 4.2 Consistency between field results and WaNuLCAS model behavior

The consistent trends between the measured and WaNuLCAS-simulated aboveground biomass (AGB) across the two years indicate that the model was accurately parameterized to represent the biophysical interactions occurring in maize sole cropping and maize–chili intercropping with hedgerow production systems. The realistic agreement between field measured and simulated values suggests that WaNuLCAS effectively captured spatial variation in maize productivity driven by similar planting positions under sole cropping conditions and hedgerows induced variations in treatments with leucaena hedgerows, thereby representing below-ground processes such as root system overlap and nutrient acquisition patterns with high reliability. This capacity to model root competition is particularly valuable because belowground interactions remain a central challenge in many crop simulation frameworks, where nutrient competition is often simplified or assumed to be homogeneous across planting rows (Banerjee et al., 2024; Wang et al., 2024).

Model simulations and field measurements jointly demonstrated that the reduction in maize biomass near the hedgerows was primarily due to nitrogen and phosphorus competition rather than water or light limitations. This finding was consistent with the observed low grain N and P concentrations and δ¹³C signatures in maize rows nearest the hedgerow, which closely resembled values from the unfertilized intercropping treatment, confirming nutrient deficiency due to competition. Meanwhile, the soil volumetric water content was consistently higher in the intercropped system (T4) than in the sole maize (T1), and both the field data and model water balance outputs indicated no water limitation. This also aligns with other studies showing that better-managed hedgerow systems can improve soil moisture storage, reduce evaporative losses, and enhance hydraulic conductivity and micro-climate on sloping upland soils (Sanchez and McCollin, 2015; Montgomery et al., 2020; Worku and Mekoya, 2025). Similarly, the maintenance of hedgerow height below the maize canopy prevented shading effects, and the light capture outputs of the model confirmed that light availability was not a limiting factor, consistent with findings that hedge height management is critical to avoid light competition in agroforestry configurations (Ladux et al., 2024; Raskin, 2025).

Nutrient management simulations further clarified how resource limitations shape system productivity over time. Doubling nitrogen while maintaining phosphorus at standard levels improved maize performance and its sustainability. In contrast, doubling phosphorus alone provided little benefit, which may be due to persistent nitrogen limitation. Combined increases in both N and P led to substantial improvements in biomass accumulation, reduced disparity between maize rows adjacent to and distant from hedgerows (row positions 1 and 2), and improved long-term production system sustainability. These results support recent evidence that balanced nutrient supplementation is necessary in mixed-species production systems where hedgerow roots exploit the same nutrient-rich upper soil layers as crops (Rao et al., 2004; Ondrasek and Zhang, 2023), and the phosphorous plays an important role in multi-cropping conditions (An et al., 2023). Moreover, adaptive fertilization that spatially aligns with nutrient demand, such as strip or banded fertilization near crop rows, has been shown to effectively mitigate competition without the need to reduce hedgerow density.

Furthermore, the integration of field measurements with WaNuLCAS-based scenario testing demonstrated that maize intercropping with hedgerows and fertilizer application systems can be productive and ecologically sustainable when nutrient competition is actively addressed. Maintaining hedgerow height below the crop canopy effectively eliminates light competition, while the absence of water limitations reflects improved microclimate buffering and soil hydraulic conditions. However, hedgerow crop root interactions may generate strong localized nutrient limitations, necessitating targeted management, such as spatially precise fertilization. These findings indicate that the WaNuLCAS model is a powerful diagnostic and decision support tool that is capable of identifying limiting resources, quantifying competition under diversified cropping conditions, and evaluating management interventions to enhance productivity and resilience. This can strengthen the role of process-based modeling in guiding smart production management under increasing environmental stress, aligning closely with global priorities for sustainable intensification and adaptive soil fertility management in upland agricultural production systems (Khongdee et al., 2022).

### 4.3 Conclusion

The results of the current study indicate that productivity differences in maize under diversified cropping conditions are primarily driven by nutrient limitations rather than water or light competition. Field experimental measurements showed low aboveground biomass, reduced grain N and P concentrations, and lower δ¹³C values in maize rows adjacent to hedgerows, indicating localized nutrient stress. These patterns were consistent with root length density assessments, which confirmed the overlap between hedgerow and maize roots in the upper soil layers (0 cm–20 cm depth). Meanwhile, the higher TDR soil volumetric water content and the absence of shading effects demonstrated that neither water nor light availability constrained maize performance in the intercropped system with hedgerows and fertilizer application.

The coherence between field observations and WaNuLCAS model outputs shows the capability of the model to accurately represent above- and below-ground competition and spatial variation in resource acquisition under diversified cropping conditions. The WaNuLCAS model supported the identification of nitrogen and phosphorus as the limiting resources and reproduced row-wise differences in aboveground biomass, as observed in the field experiments. Model-based nutrient management scenarios further indicated that a balanced increase in both nitrogen and phosphorus availability improved maize yield in rows planted adjacent to the hedgerow and enhanced overall system sustainability. These findings highlight the importance of spatially informed nutrient management in hedgerow-based intercropping systems. It is important to maintain hedgerows below the crop canopy to prevent light competition, and adequate moisture retention can be achieved through soil conservation practices in uplands. However, localized nutrient competition must be addressed through targeted fertilization to ensure yield stability in the long term.

Overall, the field model integration applied in this study provides a robust framework for diagnosing resource competition and testing management adaptations in complex agroforestry systems with varying tree densities. This approach is highly relevant for designing climate-smart upland cropping strategies, where nutrient limitations and soil degradation pose persistent challenges. The suggested site-specific fertilizer and hedgerow management can easily be managed by smallholding farmers with limited cultivated land. WaNuLCAS serves as a valuable decision-support tool for refining intercropping systems for greater productivity, resilience, and long-term sustainability.

## Data Availability

The raw data supporting the conclusions of this article will be made available by the authors, without undue reservation.
